# Implementation of Pharmacogenetics in Primary Care: A Multi-Stakeholder Perspective

**DOI:** 10.3389/fgene.2020.00010

**Published:** 2020-01-31

**Authors:** Tessel Rigter, Marleen E. Jansen, Jordy M. de Groot, Susan W.J. Janssen, Wendy Rodenburg, Martina C. Cornel

**Affiliations:** ^1^ Department of Clinical Genetics, Section Community Genetics and Amsterdam Public Health Research Institute, Amsterdam University Medical Center, Vrije Universiteit, Amsterdam, Netherlands; ^2^ Centre for Health Protection, National Institute for Public Health and the Environment, Bilthoven, Netherlands

**Keywords:** pharmacogenetics, primary care, implementation, stakeholder perspectives, qualitative research

## Abstract

**Introduction:**

Aberrant pharmacogenetic variants occur in a high proportion of people and might be relevant for the prescription of over 26 drugs in primary care. Early identification of patients who metabolize these drugs more rapidly or slowly than average could predict therapeutic effectivity and safety. Yet implementation of pharmacogenetics is progressing slowly. A high public health impact can potentially be achieved by increasing the proportion of people tested, when and where eligible according to clinical validity and utility.

**Methods:**

In this study we defined actions, roles, and responsibilities for implementation of pharmacogenetics in primary care in consultation with stakeholder groups, by using a three-step mixed-methods approach. First, to define barriers and facilitators, public pharmacists (n = 24), primary care physicians (n = 8), and patients (n = 21) participated in focus groups and face-to-face interviews. Second, a multidisciplinary expert meeting (n = 16) was organized to define desired actions, roles, and responsibilities. Third, an online Delphi Study (n = 18) was conducted to prioritize the designated actions.

**Results:**

For the integration of pharmacogenetics in primary care guidelines and practice, lack of evidence for clinical utility was mentioned as a main barrier. Furthermore, reimbursement, and facilitation of data registration and sharing were considered as key elements for future routine application of pharmacogenetic testing. Moreover, the division of roles and responsibilities, especially between general practitioners and pharmacists, is currently perceived as unclear. Sixteen actions in these four areas (clinical utility, reimbursement, data registration and sharing, and roles and responsibilities) were formulated and assigned to specific actors during the expert meeting. After ranking these 16 actions in the Delphi Study, nine actions remained pertinent, covering the four areas with at least one action. However, participants showed low agreement on the prioritization of the different actions, illustrating their different perspectives and the need to attune between them.

**Discussion:**

Stakeholders together were able to formulate required actions to achieve true integration of pharmacogenetics in primary care, but no consensus could be achieved on the prioritization of the actions. Coordination of the current independent initiatives by the different stakeholders could facilitate effective and efficient implementation of useful pharmacogenetics in primary care.

## Introduction

Pharmacogenetics (PGx) can help identify patients who metabolize certain drugs more rapidly or slowly than average in the population. Application of pharmacogenetics thereby could have substantial impact on the safety and efficacy of drugs prescribed in primary health care. In the Netherlands, more than 80 potential gene–drug pairs have been reviewed by the Dutch Pharmacogenetics Working Group (DPWG), of which 47 guidelines provide therapeutic recommendations for one or more aberrant phenotypes (Bank et al., 2018). It has been estimated that more than 95% of people have a relevant gene-variant for at least one of these drugs (Van Driest et al., 2014; Dunnenberger et al., 2015). Twenty-six of these drugs for which pharmacogenetic guidelines are available are prescribed in the primary health care setting to relatively large groups of patients (see **Supplementary Table 1**) (Houwink et al., 2015). It is therefore expected that many patients would benefit from PGx-based prescription policy (Alshabeeb et al., 2019).

**Figure1:**
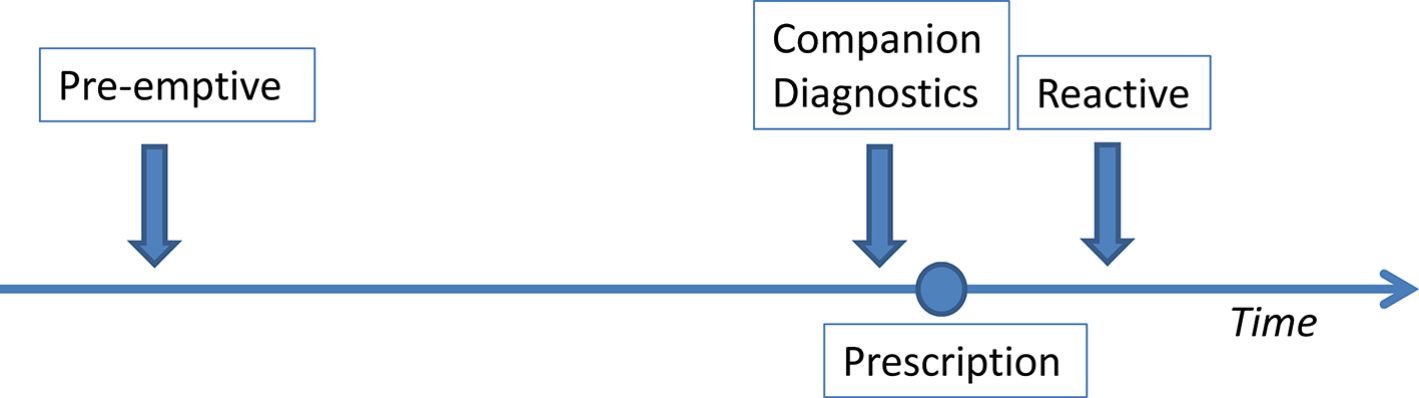
Possible timing of pharmacogenetic testing in relation to prescription.

Although expectations of PGx are high, limited application is observed in routine health care, especially in primary care (Bartlett et al., 2012; Swen and Guchelaar, 2012; Mills et al., 2013; St Sauver et al., 2016). If PGx testing is performed, it is usually done when side effects arise or when a drug lacks effectivity (i.e., reactive testing; see **Figure 1**). In secondary care sometimes testing is done before prescribing, as a companion diagnostic (CDx), for example in oncology and treatment of HIV. In a few of these cases, the Summary of Product Characteristics (SmPC) requires a PGx test to be performed before the first delivery of the medication (Weda et al., 2014). Panels are increasingly available where the most frequent and relevant variants can be tested at once (van der Wouden et al., 2017). This would allow for future prescription according to genotype for a large number of drugs. Preemptive testing, without any specific indication however, is very rare (van der Wouden et al., 2017).

Barriers and facilitators of implementation of pharmacogenetics into health care have been widely studied (Deverka et al., 2007; Swen et al., 2007; Haga and Burke, 2008; Altman et al., 2011; Ieiri, 2012; Bell et al., 2014; Perry et al., 2016; Frick et al., 2016; Hicks et al., 2016; Abbasi, 2016; Kapoor et al., 2016). Main hurdles that are described include the need for improvement in physician and pharmacist awareness and education about PGx, more insight in relevant measures for clinical validity and utility of (preemptive) PGx testing (Tonk et al., 2017; Jansen et al., 2017), and a proper infrastructure to integrate pharmacogenetics into the workflow of physicians and pharmacists (van der Wouden et al., 2017; Slob et al., 2018).

Shared initiatives to carefully plan how to overcome these barriers and draw on facilitators in Dutch primary care are limited. With this study we aimed to define actions, roles, and responsibilities for implementation of pharmacogenetics by conducting a multi-phased stakeholder study. Stakeholders such as pharmacists, primary care physicians, patients, scientists, and policy makers were invited to discuss thresholds and opportunities for next steps in the implementation of pharmacogenetics in primary care in the Netherlands. Input was collected from all relevant actors in the implementation process, from research to policy and health care. By including this range of actors, a complete view of different perspectives and expectations and broad consensus on priorities was strived for. These insights might help to formulate a strategy to progress large-scale implementation of relevant pharmacogenetics applications in routine health care, and thus contribute to a roadmap for the future.

## Material and Methods

**Figure 2:**
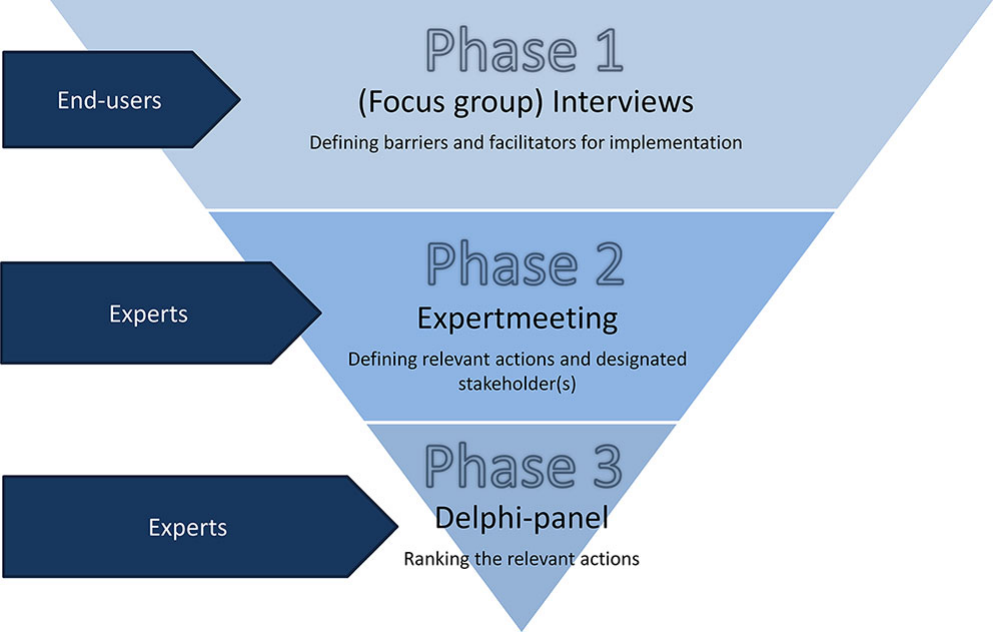
Methods and aim of the three phases of the study.

The research consisted of three phases (see **Figure 2**): 1) (focus group) interviews with end users to define barriers and facilitators for implementation of PGx in primary care; 2) an expert meeting to define necessary actions, roles, and responsibilities for responsible implementation; and 3) an online Delphi panel to prioritize these actions.

This study was approved according to the national legislation. The Medical Ethical Committee of the VU University Medical Center Amsterdam evaluated the study design and decided that the Medical Research Involving Medical Subjects Act (WMO) does not apply to this study and that further official approval is not required (2017.074).

### Phase 1: Interviews and Focus Group Interviews

To elicit perceived barriers and facilitators for implementation of pharmacogenetics in primary care, individual and focus group interviews (FGIs) were conducted with the end users: general practitioners (GPs), patients, and pharmacists.

Six FGIs were conducted: three groups with patients and three groups with pharmacists. Although a similar approach was intended for studying the views of GPs, recruitment of these participants proved unsuccessful (see **Datasheet 1** for details on recruitment and response rate of GPs). We therefore conducted eight interviews with individual general practitioners.

Purposive sampling was used to recruit the GPs, patients, and pharmacists for this study. All three key stakeholder groups were recruited from an urban environment (Amsterdam), a rural environment (Northern Limburg), and in a “mixed” region (Utrecht). The division in urban, rural, and mixed region groups was made to attract a variety of participants who would contribute to the diversity of the sample. We attempted to include community GPs and pharmacists and preferred non-experts in the PGx field to represent the average situation in current primary health care. Furthermore, we purposively invited patients who visited their GP in the last year. We did not look for specific patient groups, but for representation of GP’s patients in general. Participation was voluntarily, but when present at the (focus group) interviews, all stakeholders groups were expected to share complete perspectives, opinions, and participate actively. All stakeholders were reimbursed for travel and other expenses made for this study.

Both the interviews and focus groups were conducted using a similar semi-structured interview-guide (see **Datasheet 2**), designed to collect input on all aspects of change needed for implementation of pharmacogenetics. The interview guide followed the constellation perspective of van Raak et al. (Van Raak, 2010) [adapted by Rigter et al. (Rigter et al., 2014)], which describes that transitions in health care require new ways of doing (changes in practice), new ways of thinking (changes in culture), and new ways of organizing (changes in structure) by the actors involved. In this case, the topics included: views and expectations, required structural changes, when and whom to test, and roles and responsibilities.

The completed interviews and focus groups were anonymously transcribed verbatim and inductive content analysis was performed using thematic coding, supported by the qualitative software program: AtlasTI, version 7.5.10. The coding process was a joint effort between multiple researchers. All transcripts were individually read and coded by at least two researchers (JMdG, TR, and MJ). The findings were consistently evaluated throughout the process until consensus was reached on the coding strategy.

The official language for the interviews was Dutch; therefore, the participants’ statements were translated for use in this report.

### Phase 2: Expert Meeting

Main barriers and facilitators from the interviews were grouped into themes, which were used to organize an expert meeting to further define needed actions, roles, and responsibilities of relevant stakeholder groups. Thirty-two stakeholders with expertise in different aspects of PGx or primary care were purposively selected and invited to take part in an interactive expert meeting. Twenty-three experts accepted the invitation and 16 participated in the meeting. The following expertise were represented: health technology assessment, health care insurance and reimbursement, clinical pharmacology, clinical research, primary health care policy, patient advocacy, psychiatry, biomarker development, pharmacy, information technology in primary health care, and pharmacogenetics.

After a plenary introduction to the project and the results of the focus groups, participants were assigned to a group based on their expertise and asked to discuss a specific topic (division of responsibilities, data registration and sharing, generating evidence for guideline development, and reimbursement). Each group was chaired by a project-member who posed some pre-formulated questions (see **Datasheet 3**) to discuss and define all relevant actions and one or more designated stakeholder(s). Outcomes were summarized on a flip-over by each chair and shared between groups after the workshops to initiate a plenary discussion and formulate conclusions. Furthermore, the experts were asked to give written input if specific topics or actions were found relevant, but had not been discussed at the meeting. Based on the concluding remarks, a list of actions was formulated, serving as input for the Delphi panel.

### Phase 3: Delphi Panel

The defined actions from the expert meeting were prioritized through an online Delphi process. The Delphi technique has been a widely accepted method for data collection and reaching consensus among respondents within their domain of expertise (Dalkey and Helmer, 1963; Hsu and Sandford, 2007; Burke et al., 2009).

**Figure 3:**
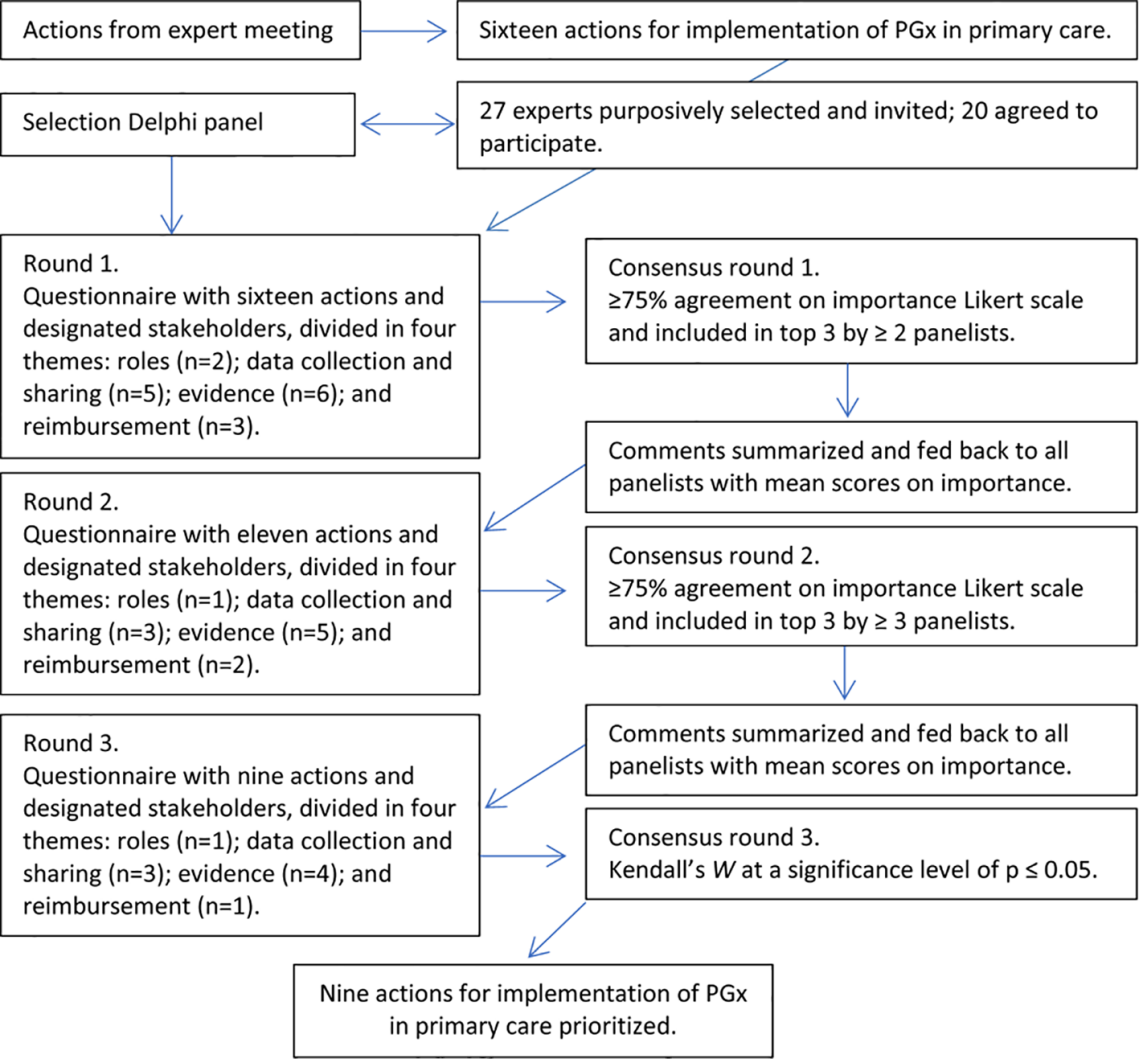
Steps of the Delphi procedure including cutoff values.

We aimed to obtain consensus of a heterogeneous Delphi panel on the prioritization of actions for implementation of PGx in primary care (see **Figure 3**). Twenty-seven experts were purposively selected and invited with similar expertise fields as the expert meeting.

Twenty experts accepted the invitation and 18 experts completed all rounds (response rate: 74.1%). Each expert e-mailed their prioritizations with arguments in three separate rounds between April and July 2017. Between rounds, all participants received an anonymized overview of answers and arguments in the next questionnaire.

The initial Delphi questionnaire contained 16 actions and suggestions for designated stakeholder(s). Participants were asked to score each action on importance on a five-point Likert scale, give a rationale for their score, and could suggest additional or different designated stakeholder(s). The questionnaire was finalized with a question to prioritize a top 3 of the actions for implementation of PGx in primary care. Criteria for consensus for each round were applied as described by Houwink et al. (Houwink et al., 2012) and Kendall’s *W* was calculated as a coefficient for concordance in the final prioritization by participants. A *p* value of ≤0.05 was considered statistically significant.

To analyze if certain experts within a group showed higher correlation in ranking the actions, participants were stratified. Each participant was allocated based on self-reported expertise. The groups were: scientists, pharmacists, policy experts, patient representatives, and GPs.

## Results

### Phase 1: Interviews and Focus Groups

Focus group interviews (FGIs) were conducted with in total 24 pharmacists and 21 patients. Unfortunately, GPs initial response rate for the focus groups was only around 1% and did not result in successful planning of a group interview (see **Datasheet 1** on recruitment and response rate of GPs), after which it was decided to conduct interviews with individual GPs. Eight GPs were interviewed. Although this approach did not allow for interactive discussions among GPs, we were able to evaluate the reasons for the low response. General lack of interest and knowledge about the topic made the input from eight individual GPs satisfactory because data saturation was reached for all three stakeholder groups. We specifically recruited non-experts from primary care (community GPs and pharmacists), but a high percentage of patients included in our study reported to have a chronic disease. For general demographics (e.g., age and years of experience in their field) of participants to phase 1, see **Supplementary Table 2**.

Relevant and recurrent themes describing barriers and facilitators for implementation of pharmacogenetics in primary care are discussed below, under headings following the main themes from the interview guide (views and expectations, organizational changes, when and whom to test, and roles and responsibilities). We have selected quotes to illustrate the views and arguments within these themes.

#### Views and Expectations

In the (focus group) interviews, GPs and pharmacists expressed that pharmacogenetics is currently rarely considered or used by GPs.

“To me it [PGx testing] is all very new [… ], I don’t think about it [PGx testing]. This totally isn’t something that I am considering as a GP.” GP5, 5:54

Patients themselves said to generally be unaware of (potential usefulness) of the influence of genes on drug response.

“I am surprised by the list of drugs [you just showed] for which they know they could work differently for certain groups of people.” Patient FG5, 1:30

Some pharmacists said to have experience with pharmacogenetics in their practice, either by being involved in a pilot study or responding to (anecdotal) evidence of utility of PGx testing for specific drugs.

“[In the context of a PGx implementation study] it is a small group of patients still, fifty now, that we have genotyped.” Pharmacist, FG3, 3:3

“[after a year of raising awareness of PGx for clopidogrel] I have to say: [… ] it has been more than a year, we only have five contra-indications registered on 40,000 patients.” Pharmacist, FG3, 4:37

Although most participants of the interviews seemed to recognize the potential of pharmacogenetics—to reduce adverse drug reaction, increase effectiveness of treatment, and possibly indirectly increase adherence—not all seemed convinced of the urgency to press large-scale implementation. Especially general practitioners were perceived as reluctant to change their current practice of “trial-and-error” when prescribing drugs.

“In [current] practice they [GPs] will just play with the [medication] dose: we will increase it and see what happens, decrease it and if drug A doesn’t work, we will try drug B [… ]. It never really comes to the test. Even though that is the most likely cause of the problem.” Pharmacist, FG1, 3:35

“In general our profession is relatively conservative when it comes to new developments: first seeing what the effects are and what we gain from it and what the outcomes are and then getting on board. There are few people who then are pioneers [… ]” GP8, 10:3

Especially pharmacists seemed supportive of the use of pharmacogenetics and were expecting more applications to be developed to optimize treatment for the patient. It was also expressed that it could be an opportunity to expand the current job responsibilities and accompanying funding structure of pharmacists. Consequently, most pharmacists showed disappointment about the current lack of use of the potential of PGx in primary care.

“I think that more should be done with it [PGx] and that you should not wait until people develop all sorts of, euhm, just muddle along with their drugs. That we should be more pro-active.”Pharmacist, FG3, 5:2

Although most participating pharmacists said to have both the knowledge and infrastructure available to increasingly start applying pharmacogenetics in daily practice, there was doubt as to whether their peers would be as well-equipped.

It was acknowledged by both GPs and pharmacists that there currently was a lack of knowledge and clear protocols for effective implementation of pharmacogenetics in primary care, in particular for GPs.

#### Organizational Changes

Lack of evidence on clinical utility was mentioned as a general barrier to include pharmacogenetic dosing advices in guidelines for general practitioners.

“[… ] As long as you don’t know the effectiveness, but also the costs and benefits in primary care. I would think, that as a GP, you should be very careful in this matter.” GP5, 5:16

Besides lack of evidence and easily accessible guidelines, other main structural prerequisites were mentioned, such as reimbursement of the test and subsequent therapy, user-friendly software systems, and data sharing infrastructures.

“It should be clear, practical and applicable, otherwise it won’t happen.” Pharmacist, FG3, 5:29

Another impediment to the routine application of pharmacogenetics surfaced when discussing reimbursement. It was expressed that potentially investments are required in a different silo of health care than where the return on investment will appear.

Efficient data exchange was mentioned by all participant-groups as a prerequisite for effective implementation. This included exchange of guidelines, since participants expressed that existing pharmacogenetic dosing advices are only included in routine health care guidelines for pharmacists, but are not easily accessible for general practitioners. Moreover, exchange of test results between GPs and pharmacists, but also between professionals in primary and secondary care, was requested to prevent unnecessary repeated testing.

Furthermore, protocols when to test a patient (see **Figure 1**) are considered essential to implement pharmacogenetics successfully.

“You should know: when do you want a test? Do you want it before therapy or when the therapy doesn’t work or when adverse reactions occur? Who will you test?” Pharmacist, FG1, 4:97

#### When and Whom to Test

When discussing the best timing of testing, there seemed to be a tendency to prefer preemptive testing because of the direct usefulness of the information at the moment of prescription of a relevant drug.

“I think something is going to change [… ] and that you will advise more proactively instead of reactively. Because that is a profile that is established since moment zero [… ], then you already know for the coming years what your patient is allowed to have and what not.” Pharmacist, FG2, 1:166

“The moment of testing… I think in the future we will go towards the moment a baby is born, that immediately a DNA-profile is made.” Patient, FG6, 4:2

However, there was no consensus about the target population (e.g., newborns or specific subgroups later in life) and questions arose about the (cost)-effectiveness of preemptive testing. Therefore, some participants preferred companion diagnostic or reactive testing.

“But if they are not going to use drugs, then there is no need to know it. You can also wait until the moment someone is going to use drugs.” Pharmacist, FG3, 5:62

“I would still argue to do it on indication alone [… ], so if you expect problems, but not standard with everybody.” GP6, 7:56

Deciding on most appropriate timing of testing proved complex and therefore participants expect it to be resolved at policy level, as well as clearly described in protocols.

#### Roles and Responsibilities in Applying PGx

Disagreement exists about the best division of responsibilities between general practitioners and pharmacists, and the patient’s role. GPs generally expressed the desire to be able to request the test themselves and want to remain end-responsible for the correct dosing of drugs. GPs mainly see the role of the pharmacist as signaling and advising on drug-prescription, including pharmacogenetic influences.

“[… ] I expect the pharmacist to know more than I know from pharmacokinetics and that sort of things and that he could advise me better in: this combination should be avoided in any case and this can go together.” GP8, 10:2

Pharmacists themselves seem to picture a more central role in pharmacogenetics for their profession; some even as party responsible for all prescription of drugs in general.

“But in that case I would actually want the doctor to only write down the diagnosis. [… ]. And that I come up with the pills for that.” Pharmacist, FG2, 1:167

However, pharmacists generally also seem to acknowledge that this role should be granted by GPs as well as patients.

Patients explicitly prefer the GP as having the final responsibility and being the contact person when it comes to applying pharmacogenetics, mainly because of familiarity and trust.

“But I think a pharmacist in itself, is too commercial to do such things [order a PGx test and adjust treatment accordingly]. A blood drawing station or so [could do that], okay, or the GP himself, but a pharmacist absolutely not.” Patient, FG6, 10: 6

All participants emphasize that there is a need for cooperation and explicitness about roles and responsibilities between GPs and pharmacists.

“Together [the GP and the pharmacist] we can make sure that the chosen therapy gets a very good chance of success when it, ehm, when the genotypes of the patient are known.” Pharmacist, FG1, 4:20

To maintain the relationship of trust and give all stakeholders the time to become acquainted with the new division of roles and responsibilities, participants mentioned that it would be wise to not act precipitately and implement pharmacogenetics in phases.

In order to list all required actions for implementation of PGx, output from the interviews was used to organize an expert meeting in the next phase of the study.

### Phase 2: Expert Meeting

Based on the interview data, four themes were defined and discussed in an expert meeting: 1) division of responsibilities; 2) data registration and sharing; 3) generating evidence for guideline development; and 4) reimbursement. During the expert meeting, actions within these themes were formulated, with an indication of the responsible stakeholders for the action (see **Table 1**).

**Table 1:** Actions, roles, and responsibilities as discussed in the expert meeting.

Themes	Actions	Responsible stakeholder(s)
Division of responsibilities	*Develop a national guideline on collaboration.	*Health care provider organizations of pharmacists and GPs (KNMP/NHG).*
	*Make agreements on a regional level about when and who can request PGx tests.*	*Regional groups for pharmacotherapeutic consultation (local organization of GPs and pharmacists).*
Data registration and sharing	*Define relevant data that should be registered and shared between health care professionals for effective use of PGx.	*Health care provider organizations of pharmacists and GPs (KNMP/NHG).*
	*Standardize patient data that needs to be registered with regard to PGx.	*Health care provider organizations of pharmacists and GPs (KNMP/NHG) and NICTIZ (National IT Institute in Health Care).*
	*Further develop the National Link Point to enable easy exchange of PGx data between health care professionals.*	*VZVZ (Association of health care providers for health communication) at the initiative of the health care provider organizations (KNMP/NHG) in collaboration with NICTIZ (National IT Institute in Health Care).*
	*Facilitate aligned registration for the reason of adjusting a patient’s treatment regime, to monitor and evaluate effectiveness of applying PGx.*	*NICTIZ (National IT Institute in Health Care), in collaboration with software developers HIS/AIS (information systems for GPs/pharmacists), at the initiative of the Dutch GP association (LHV)/Royal Dutch Pharmacists Association (KNMP).*
	*Adjust or develop software systems to facilitate applying PGx.*	*Software developers HIS/AIS (information systems for GPs/pharmacists), at the initiative of the Dutch GP association (LHV)/Royal Dutch Pharmacists Association (KNMP), in collaboration with NICTIZ (National IT Institute in Health Care).*
Generating evidence for guideline development	*Gather data on the number of prevented ineffective or adverse drug responses through PGx.	*Funders for research/independent research institutes/scientific organizations.*
	*Validate the predictive value of PGx tests through prospective or observational research.	*Scientific organizations.*
	*Assess the cost saving of PGx test through pharmaco-economic studies.	*Scientific organizations.*
	*Collect data on the impact on clinical outcomes by assessing the patient experience of the severity of ineffective or adverse drug response.	*Scientific organizations, together with patient organizations.*
	*Develop aligned patient information on the benefit of PGx tests. Monitor data on the frequency of genetic variants that are tested with PGx.	*Health care provider organizations (KNMP/NHG) of pharmacists and GPs together with patient organizations Independent research institutes.*
	*Monitor data on the frequency of genetic variants that are tested with PGx.*	*Independent research institutes.*
Reimbursement	*Include PGx tests as an optional test for general practitioners in their guideline.	*Dutch organization for general practitioners (NHG).*
	*Develop aligned patient information on the costs of PGx test and the impact on their health care insurance reimbursement.*	*Health care provider organizations of pharmacists and GPs (KNMP/NHG), in collaboration with ZN (Dutch Health Care Insurers) and patient organizations.*
	*Define and prioritize disease areas eligible for reimbursement based on data on clinical utility.*	*Health insurers and ZINl (Dutch Health Care Institute).*

Statements preceded by an asterisk (*) remained after three iterations of the Delphi procedure. PGx pharmacogenetics, GP general practitioner.

### Phase 3: Delphi Panel

The formulated actions and responsibilities were prioritized by a heterogeneous Delphi panel in the third phase of the study. Eighteen out of 20 experts in the Delphi panel completed all rounds. Ten experts were female (50%), and the mean age was 48.5 years (SD = 9.9). We aimed to include representatives of key stakeholders and similar expertise as in the expert meeting, but, mainly due to time constrains, some expert groups allocated this task to another colleague. Ten of the panelists also participated in the expert meeting. From the 16 actions suggested during the expert meeting, nine remained after the three iterations of the Delphi procedure (see **Table 1**).

In the overall analysis, results showed low agreement between participants on the ranking of the remaining nine actions (*W* = 0.14, *p* = 0.011; see **Supplementary Table 3**) While not statistically significant, the highest correlations in ranking were seen between respondents within the expertise pharmacists (*W* = 0.617, *p* = 0.275) and GPs (*W* = 0.842, *p* = 0.097). The participants within these two groups show moderate agreement on the ranking of the actions, but—as can be deducted from the overall analysis—the ranking differs between the groups. For example, on average, action 15 “Include PGx tests as an optional test for general practitioners in their guideline” was ranked third of nine by pharmacists and 8.5 (i.e., almost last place) by GPs.

High consensus on a topic’s importance did not always translate into many experts putting it in their top 3, and vice versa (see **Supplementary Table 4**). In round 1 for example, only 75% of the panelist scored the statement “*Validate the predictive value of PGx tests through prospective or observational research*” as (very) important, while 7 of the 20 panelists put the statement in their top 3. In contrast, while 95% of panelist scored the statement “*Standardize patient data that needs to be registered with regard to PGx*” as (very) important, only three put the statement in their top 3. In support of this last statement, some panelist argued that “From my point of view, this is one of the major barriers” and “Without standardized data management, appropriate and useful application of pharmacogenetics is not possible.”, while others also mentioned that they thought “Are all patient data not already standardized? Seems logical to do so.”, suggesting that some panelist scored statements lower because they assumed the action was already in place.

Looking at statements that were accepted in the first round, but then rejected in the second round (*n* = 2), the statement “*Define and prioritize disease areas eligible for reimbursement based on data on clinical utility*” dropped from 75% consensus on importance to 56%. While in round 1 supportive panelists mentioned “Start with diseases that have the most impact and/or prevalence” and “Start with disease areas that seemingly will have the highest clinical utility,” others stated that “To be able to prioritize, you need the research data mentioned in the other statements.” or “Patient characteristics and individual response or type of medication are more important than disease areas.”, which may have led to other participants changing their scores.

The nine statements that remained after three iterations of Delphi procedure also had differing arguments from panelists why an action was or was not important. For example, the action “*Develop a national guideline on collaboration*” was considered important because “It is essential that it will become clear who will lead the way, who is responsible in daily practice, and how it will be implemented.”, while another panelist stated that PGx should be “included in general collaboration guidelines, not a specific one for pharmacogenetics.” While many of the panelist considered the actions under evidence important, because “If there is no clinical utility, then the other actions also become less important” and “First research, then implementation,” one panelist was skeptical “Gathering data on prevented ADRs is wrongly considered as very important, it should be less prominent.” and considered collecting data on the patient experience from side effects “Unethical. We have a classification system for ADRs.” Informing the patient was also considered highly relevant action, as one panelist stated “Honest and independent patient information that is also available online seems necessary to me.” Some panelist fed back that they missed an action to educate GPs.

Overall, the Delphi procedure helped to define nine actions that were considered important by most experts. The majority of the actions (five out of nine) fall within the category of generating evidence for guideline development, indicating that this is currently perceived as a main barrier. However, no consensus on which of the actions should be top priority was reached among the Delphi panelists.

## Discussion

With this study, we aimed to define actions, roles, and responsibilities for implementation of pharmacogenetics in primary care. Based on a qualitative inventory of perceived barriers and facilitators for responsible implementation of pharmacogenetics among primary care end users in the first phase of this study, experts formulated and ranked actions to achieve effective application in the two later stages (see **Figure 2**). The (focus group) interviews, as well as the input from expert meeting, indicate that currently the main barrier for implementation is the lack of insight into clinical utility of pharmacogenetics testing. Some stakeholders express they are convinced of the need to use pharmacogenetic information in primary care, but others state that necessary evidence for preemptive testing in primary care is lacking. Current publications give little insight in the actual (cost-)effectiveness of a structural offer of pharmacogenetics testing and in what context it could prove most beneficial to patients. Although evidence on what to do in case of specific phenotypes has been translated into guidelines, evidence of how to generate and use these genotypes in primary care is lacking. This is partly due to uncertainty which patients to test at which time point. This issue is subject to recent discussions: if there is no clear view of the actual context of testing, researchers will keep failing at providing insight into relevant measures for policy decisions and stick to reporting associations between drugs and genotypes (Tonk et al., 2017; Jansen et al., 2017).

If clinical utility is established however, for example from results from current studies on implementation of preemptive pharmacogenetics panels [e.g., the uPGx project: (van der Wouden et al., 2017)], experts involved in this study acknowledge that there are still other barriers to overcome. The required actions involve making clear arrangements for collaboration between different stakeholders, data registration and sharing, and reimbursement of testing and follow-up.

It is noteworthy that awareness and education among (primary) healthcare professionals on PGx has not surfaced as a main topic requiring action in our study. Many recent publications have described awareness and education as important prerequisites for implementation. Different efforts have therefore focused on developing (continuous) education programs for professionals (Just et al., 2017). When asked to formulate actions, experts in our study, however, expressed other prerequisites instead of awareness and education as such, perhaps because other actions are considered more urgent. A clear example is the prerequisite to construct guidelines and protocols on when and whom to test, and the need for evidence which could be incorporated in professional guidelines. Creating awareness and effective education will have to build on these guidelines and protocols.

Although this study provides insight into the actions required by different stakeholders to achieve true integration of pharmacogenetics in primary care, there was no consensus on the priority of each action. This might be due to a lack of a collective sense of urgency to adopt this innovation in daily practice and/or the multitude of stakeholders that are expected to take action. In spite of the fact that some stakeholders did seem to perceive their actions as urgent, collaboration between stakeholder groups was scarce. Furthermore, the incentives for the different stakeholders to undertake the actions described seem to be unclear or perhaps even lacking. There seems to be no (independent) coordination of the initiatives that contribute to the required actions for effective and efficient integration of pharmacogenetics in primary care, perhaps leading to suboptimal attuning between stakeholders.

Strengths of this study include the fact that the stakeholders themselves defined actions and priorities. This contributes to the likelihood that relevant and feasible actions towards implementation of PGx in primary care were defined and could help in raising awareness about the required steps. Eventually, this could perhaps motivate the professionals to take action. Furthermore, the different methods used in this study provided a platform for the different stakeholder*s* to share their views on how to take the use of pharmacogenetics in primary care to a next level. For the Dutch health care setting, and potentially other countries as well, this might therefore be a good model towards finding consensus on who is expected to undertake which responsibilities.

To ensure internal validity of the study, researcher triangulation was adopted for the coding and interpretation of the data: multiple researchers from different backgrounds were involved.

It is possible that outcomes of this study cannot be fully translated to other countries because of the Dutch context, involving specific data infrastructures and, e.g., the particular role of the GP as a gatekeeper in the Dutch health care system. Although we attempted to include non-expert GPs, patients, and pharmacists from different regions in the Netherlands (both from cities and more rural areas) to increase transferability of the results, it should be noted that especially the pharmacists and GPs included in the (focus group) interviews expressed that they might be more interested or knowledgeable about PGx than the general pharmacist/GP and/or patient. This might imply even more thresholds in real life, such as a high proportion of stakeholders who are unknowingly unable.

GPs proved difficult to motivate to participate in our study, with a response rate for the intended focus groups of around 1%. This is comparable with response rates from GPs in other studies on (pharmaco)genetics [e.g., a focus group study with response rate of 0.45% by Jans et al. (Jans et al., 2013) and a questionnaire survey with a response rate of 3% by Stanek et al. (Stanek et al., 2012)]. GPs that participated to our interviews explained that the lack of interest most likely relates to the unfamiliarity and lack of knowledge on the topic.

## Conclusion and Future Perspective

For innovations to be sustainably integrated in health care, it is known that changes in culture, structure, and practice are required (Van Raak, 2010; Rigter et al., 2014; Holtkamp et al., 2017). The stakeholders in this study were able to define specific actions on all these levels to pave the road for integration of pharmacogenetics into primary care. Participants showed low agreement on the ranking of priorities for the different actions.

**Figure 4:**
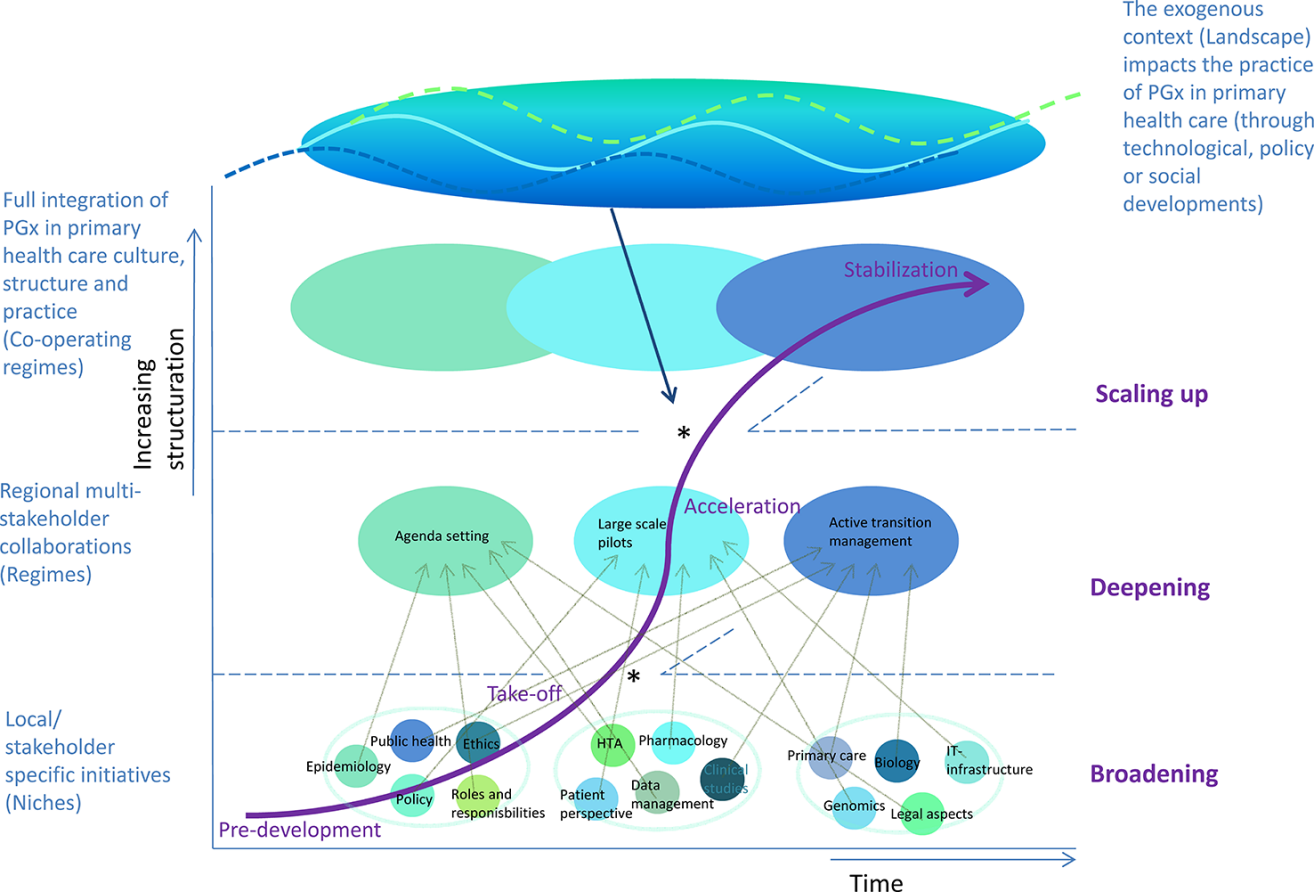
True integration of pharmacogenomics in primary health care requires different transitions [adapted from (Rotmans et al., 2001; Geels and Schot, 2002; Geels, 2007)]. Local or stakeholder-specific initiatives will need to transform to a patchwork of multi-stakeholder collaborations which could create pressure to sustainably change the existing health care culture, structure, and practice. This could be achieved by joint efforts to 1) broaden implementation: transitioning from the pre-development to take-off phase of transitions requires effective learning processes on multiple dimensions; 2) deepening implementation: transitioning from the take-off phase to acceleration of transitions requires attunement and collaboration between stakeholders to align (lessons) from earlier niche applications; and 3) scaling-up implementation: transitioning from the acceleration to stabilization phase of transitions requires true changes in thinking, organizing, and doing of stakeholders. Furthermore, taking advantage of windows of opportunities (*) to next phases in transition (often achieved by alignment of different initiatives and/or stakeholders) could facilitate structuration and thereby integration of new ways of thinking, doing, and organizing.

Different stakeholder groups have taken initiative (to prepare) for some of the prerequisites that have been formulated in this study, but there is still a lack of a collective driver of change. From a transition management perspective, it seems some aspects of implementation are deepened in the current niche initiatives (at a micro-level), but these are not substantially broadened to eventually achieve scaling-up to full implementation in primary care (Rotmans et al., 2001; Geels, 2002; Geels FW, 2007). This might be due to a lack of coordination of the different actions in the field and eventually might lead to stagnation of structuration of initiatives. **Figure 4** shows an overview of the implementation process for PGx in primary care, from a transition management perspective. The model summarizes general transition phases and aspects. Based on existing transition management models, the figure provides insight into the needs for full integration of PGx in primary health care culture, structure, and practice.

As shown in **Figure 4**, there seems to be a window of opportunity in the current awareness of the potential of pharmacogenomics under researchers, policy makers, and health care professionals, as well as the eagerness of public pharmacists to use PGx information in their prescription practice. Without a collective effort to substantially change current culture, structure, and practice however, implementation of PGx in primary care might not answer to the needs of stakeholders, resulting in fading enthusiasm and potentially even decreasing trust in effectiveness of PGx. Missing this window of opportunity might thereby lead to premature plateau in the curve representing “lock-in” or even a backlash in transition (v.d. Brugge and Rotmans, 2007).

If stakeholders want national adoption of pharmacogenetics testing in primary care to be a success, we suggest that champions with good examples of effective application engage the field, including funding agencies (in science as well as care). Probably recent initiatives in secondary care could be used for this purpose: e.g., applications of PGx in psychiatric care and oncology, but also opportunistic screening for PGx variants in exomes sequenced for diagnostic purposes. This requires early involvement of stakeholders from primary care to discuss implications for their practice. Furthermore, developments in the data infrastructure in (primary) health care could facilitate adoption of PGx information in patient care. An alternative suggestion is to allocate top-down funding at a policy level for resources for clinicians and scientists to support collaboration and stimulate implementation of PGx in health care, similar to the IGNITE Initiative (funded by the NIH) in the USA (Geels FW, 2007) or embedded in a national initiative to foster implementation of genomic medicine, similar to the Genomics England (mainly funded by NHS England and the National Institute of Health Research) in the UK (Website Genomics England, ). This could facilitate national cooperation and more efficient broadening and scaling up of initiatives that are currently undertaken mostly at regional or professional-subgroup level. Perhaps most importantly, a collective drive to collect evidence of clinical utility of PGx testing will have to be achieved to substantiate (ethical) evaluation of the impact of PGx and ensure its responsible and sustainable implementation.

## Data Availability Statement

Anonymized data generated for this study is available on request to the corresponding author.

## Author Contributions

TR, WR, MJ, and MC worked together in setting the study objectives and designing the project. SJ and MC suggested the main concept about the project and highlighted its importance. TR and MJ took the lead in collecting the data, with help from JG in the interview phase and regular feedback of WR and MC. TR drafted the main part of the manuscript, with specific input of MJ for the parts on the Delphi Study. All co-authors reviewed and modified the paper.

## Funding

This research was funded through RIVM Strategic research project “Personalised Medicine, Eligible or not?” (S/132001) and was partly executed at the Section of Community Genetics, Amsterdam UMC/VU University Medical Center.

## Conflict of Interest

The authors declare that the research was conducted in the absence of any commercial or financial relationships that could be construed as a potential conflict of interest.
