# Supplementary material for: Implementation of Pharmacogenetics in Primary Care: A Multi-Stakeholder Perspective
Source: Front Genet. 2020 Jan 31;11:10. doi: 10.3389/fgene.2020.00010 (PMC7006602; doi:10.3389/fgene.2020.00010)
Supplement: Supplementary file 1 [file DataSheet_1.pdf]

Supplementary information recruitment and response rate of GPs

We applied for accreditation for all focus groups with professionals (pharmacists as well as general practitioners). Unlike the focus groups for the pharmacists, the accreditation for the focus groups with GPs was not granted. We therefore decided to compensate GPs for their participation with a 100,-euro voucher (as well as reimbursing travel expenses).
Around 30 general practitioners out of our own direct network were informed about the study via e-mail or telephone.
Furthermore, notifications about the research were distributed by postal mail, e-mail and through publications in regional (online and printed) GP newsletters, via local GP associations. These publications were drafted with help from the target group and gave a short description of the study, contact details and specifically mentioned compensation.
Moreover, around 1000 flyers had been printed and distributed at a national GP congress (Landelijke Huisartsenbeurs Utrecht, April 2, 2016), simultaneously giving the opportunity to participate in a focus group session that same day at a location across the street.
Eventually 5 focus groups were planned in 4 different regions. In total around 1000 GPs have been directly informed about the study and were invited to participate. 12 GPs were found willing to participate (a response rate of around 1%), making the groups too small to have effective focus group interaction. Therefore it was decided to invite them for individual (face-to-face or telephone) interviews. A total of 8 GPs were interviewed.
