# Supplementary material for: Implementation of Pharmacogenetics in Primary Care: A Multi-Stakeholder Perspective
Source: Front Genet. 2020 Jan 31;11:10. doi: 10.3389/fgene.2020.00010 (PMC7006602; doi:10.3389/fgene.2020.00010)
Supplement: Supplementary file 2 [file DataSheet_2.pdf]

Supplementary information: Semi-structured interview-guide

As a general guide the following themes/questions were addressed during the (focus group) interviews (translated form Dutch). Depending on the background of the interviewee(s) minor adaptations have been made.

Introduction

- *Welcome*
- *Introduction interviewer/chair (and assistant)*
- *Background study:*
    - *Introduction of the background and the goals of the interview/focus group (including introduction of concepts pharmacogenetics and personalized medicine)*
    - *Explanation of procedure during and after (focus group) interview*
    - *Clarification of expectations to explain to the interviewee/FG participants:*
        - *Invitation to discuss/share opinions and views/suggestions*
        - *Questions will be posed to respond to, no obligation*
        - *Role interviewer and assistant (minutes secretary/observer)*
    - *Short introductions with (background and reason to participate of) interviewee/FG participants*

Questions

- *Opening:*
    - *(Write down) what comes to your mind when you think about pharmacogenetics (in your daily work)?*
        - *a) Where is it currently applied?*
        - *b) How is it currently applied?*
- *Vision:*
    - *How do you see application of pharmacogenetics in primary care in the next 5 years?*
        - *a) Where is it applied?*
        - *b) With what purpose?*
- *Advantages/disadvantages:*
    - *What advantages are there for application of pharmacogenetics in primary care?*
    - *What disadvantages are there for application of pharmacogenetics in primary care?*
- *Needs:*
    - *What would you need to apply pharmacogenomics in a sensible way (if needed, give examples such as: information, organization, actors, your own role)*
- *Procedure/roles and responsibilities:*
    - *Can you the describe you ideal procedure for application of pharmacogenetics in primary care?*
        - *a) Information-exchange:*
            - *What information is relevant?*
            - *How should that information be provided (orally/written/online etc.)?*
            - *When should this information be provided?*
            - *Where would you want to go if you have further questions?*
        - *b) Organisation:*
            - *When should the test be done?*
            - *Where is the sample (blood/saliva) taken?*
            - *How long would you want to wait for the results?*
            - *What happens after the test-results?*
            - *Who pays for the test (and how much does it cost maximally)?*
        - *c) Health care providers involved:*
            - *Who requests the PGx test and when?*
            - *Who is giving information abt. the test?*
            - *Who interprets the test results?*
            - *Who discusses the results of the test (with whom)?*
            - *Does the patient receive other information (e.g. pamphlet/gene-profile on a card)?*
            - *What is role of pharmacists?*
            - *What is role of GPs?*
            - *What is role of the lab?*
        - *d) Role of the patient:*
            - *How do you see the role of the patient?*
                - *Consent of the patient?*
                - *Are pharmacogenetics profiles determined on patients request?*

- *Implementation:*
    - *Which factors are relevant for implementation of pharmacogenetics in primary care?*
    - *Which factors inhibit implementation of pharmacogenetics in primary care?*
- *Conclusion:*
    - *The goal was to elucidate your views and expectation about the implementation of pharmacogenetics in practice. Is there anything that has not been discussed, but requires further attention?*
