# Supplementary material for: Implementation of Pharmacogenetics in Primary Care: A Multi-Stakeholder Perspective
Source: Front Genet. 2020 Jan 31;11:10. doi: 10.3389/fgene.2020.00010 (PMC7006602; doi:10.3389/fgene.2020.00010)
Supplement: Supplementary file 3 [file DataSheet_3.pdf]

Expertmeeting 13 februari 2017: “Personalized medicine: eligible or not?”
*About eligibility of pharmacogenetics for primary care*

# Theme: Evidence for guideline development

## Group A – questions and notes

Workshop 1: 13:00 – 14:00

| What measures/numbers/data/evidence are needed for guideline development? |
|---|
| |

| How should these be studied? |
|---|
| |

| Who is responsible for studying and communicating the different outcome measures? |
|---|
| |

Rijksinstituut voor Volksgezondheid en Milieu
*Ministerie van Volksgezondheid, Welzijn en Sport*

VUmc VU University Medical Center Amsterdam

Expertmeeting 13 februari 2017: “Personalized medicine: eligible or not?”
*About eligibility of pharmacogenetics for primary care*

# Theme: Financing and reimbursement

## Group A – questions and notes

Workshop 2: 14:30 – 15:30

| Can you think of a reimbursement structure for a panel of pharmacogenetic-genes? What are prerequisites? |
| --- |
| |

| Who should be able/be responsible for requesting pharmacogenetic tests? And when/under what circumstances? |
| --- |
| |

| How can clear and functional arrangements about reimbursement of pharmacogenetic tests be made? |
| --- |
| |

Rijksinstituut voor Volksgezondheid en Milieu
*Ministerie van Volksgezondheid, Welzijn en Sport*

VUmc VU University Medical Center Amsterdam

# Theme: Roles and responsibilities of health care providers

## Group B – questions and notes

Workshop 1: 13:00 – 14:00

| How could consensus be reached about the roles and responsibilities concerning pharmacogenetic care of direct health care providers. |
|---|
| |

| How can we realize clear definition of the role of e.g. the clinical chemists and the patient? |
|---|
| |

| What agreements should be made about roles and responsibilitiea? |
|---|
| |

| Who is responsible for facilitating these agreements? |
|---|
| |

Expertmeeting 13 februari 2017: “Personalized medicine: eligible or not?”
*About eligibility of pharmacogenetics for primary care*

# Theme: Registering and exchanging patient data for health care providers

## Group B – questions and notes

Workshop 2: 14:30 – 15:30

| What are prerequisites for a user friendly registration and exchange system? |
|---|
| |

| What data should be registered and exchanged? Between whom? |
|---|
| |

| What are risks of this data registration and exchange? |
|---|
| |

| How can we achieve safe registration and exchange? |
| --- |
| |

| Who is responsible for development and monitoring of this user friendly registration and exchange system? |
| --- |
| |
