# Supplementary material for: Implementation of Pharmacogenetics in Primary Care: A Multi-Stakeholder Perspective
Source: Front Genet. 2020 Jan 31;11:10. doi: 10.3389/fgene.2020.00010 (PMC7006602; doi:10.3389/fgene.2020.00010)
Supplement: Supplementary file 4 [file Table_1.docx]

Supplementary Table 1: Drug-gene-interactions potentially relevant to primary care (adapted from: *Houwink EJF et al. Farmacogenetica in de eerstelijnszorg: toepassing en toekomstverwachtingen. NTvG. 2015; 159:A9204*)

| **Drug** | **Gene** |
| --- | --- |
| Flucloxacillin | HLA-B |
| Phenprocoumon | VKORC1 |
| Acenocoumarol | VKORC1 |
| Metoprolol | CYP2D6 |
| Lansoprazole | CYP2C19 |
| Esomeprazole | CYP2C19 |
| Omeprazole | CYP2C19 |
| Pantoprazole | CYP2C19 |
| Codeine | CYP2D6 |
| Oxycodone | CYP2D6 |
| Tramadol | CYP2D6 |
| Amitriptyline | CYP2D6 |
| Clomipramine | CYP2D6 |
| Doxepin | CYP2D6 |
| Imipramine | CYP2D6/CYP2C19 |
| Nortriptyline | CYP2D6 |
| Paroxetine | CYP2D6 |
| Venlafaxine | CYP2D6 |
| Citalopram | CYP2C19 |
| Escitalopram | CYP2C19 |
| Sertraline | CYP2C19 |
| Haloperidol | CYP2D6 |
| Risperidone | CYP2D6 |
| Clozapine | CYP1A2 |
| Atorvastatin | SLCO1B1 |
| Simvastatin | SLCO1B1 |
