# Supplementary material for: Implementation of Pharmacogenetics in Primary Care: A Multi-Stakeholder Perspective
Source: Front Genet. 2020 Jan 31;11:10. doi: 10.3389/fgene.2020.00010 (PMC7006602; doi:10.3389/fgene.2020.00010)
Supplement: Supplementary file 5 [file Table_2.docx]

Supplementary Table 2: General demographics of participants in phase 1

|  | Total participants (n) | Female gender n (%) | Age, years (mean) | Experience, years (range (mean )) | Received education on PGx, n (%) | Patient with chronic disease, yes (%) |
| --- | --- | --- | --- | --- | --- | --- |
| GPs | 8 | 5 (62,5%) | 31-63 (48) | 1-35 (21) | 3 (37,5%) | N/A |
| Pharmacists | 22 | 12 (54,5%) | 25-65 (48) | 1-40 (21) | 6 (40%) | N/A |
| Patients | 19 | 4 (21%) | 17-68 (44) | N/A | N/A | 13 (67%) |
