# Supplementary material for: Implementation of Pharmacogenetics in Primary Care: A Multi-Stakeholder Perspective
Source: Front Genet. 2020 Jan 31;11:10. doi: 10.3389/fgene.2020.00010 (PMC7006602; doi:10.3389/fgene.2020.00010)
Supplement: Supplementary file 6 [file Table_3.docx]

Supplementary Table 3: Mean rank order and Kendall’s W per action in the resultant nine after three Delphi rounds

|  | **Mean rank order** | | | | | | |
| --- | --- | --- | --- | --- | --- | --- | --- |
|  | **Overall** | **Scientists** | **Pharmacists** | **Policy** | | **Patients** | **GPs** |
|  |  |  |  | Pharmacists | GPs |  |  |
| 1. National collaboration guideline | 5.44 | 5.00 | 7.00 | 4.60 | 3.67 | 9.00 | 8.00 |
| 3. Define relevant data | 5.11 | 5.00 | 2.00 | 5.40 | 6.00 | 5.00 | 6.50 |
| 6. Standardize patient data | 3.39 | 3.20 | 3.50 | 3.80 | 3.33 | 4.00 | 2.50 |
| 8. Gather data prevented events | 3.83 | 3.40 | 3.50 | 4.60 | 4.33 | 6.00 | 1.50 |
| 10. Validate predictive value | 4.67 | 5.20 | 6.00 | 5.00 | 4.33 | 3.00 | 2.50 |
| 13. Patient information on PGx benefit | 4.83 | 4.60 | 8.50 | 3.80 | 3.00 | 7.00 | 6.00 |
| 11. Pharmaco-economic research | 6.00 | 6.60 | 6.50 | 5.20 | 7.67 | 2.00 | 5.50 |
| 12. Impact clinical outcome | 5.00 | 4.60 | 5.00 | 6.20 | 5.67 | 1.00 | 4.00 |
| 15. Optional in GP guideline | 6.72 | 7.40 | 3.00 | 6.40 | 7.00 | 8.00 | 8.50 |
| *Kendall’s W* | *0.138* | *0.241* | *0.617* | *0.113* | *0.367* | *N=1* | *0.842* |
| *p-value* | *0.011* | *0.290* | *0.275* | *0.806* | *0.359* | *N=1* | *0.097* |
