# Supplementary material for: Implementation of Pharmacogenetics in Primary Care: A Multi-Stakeholder Perspective
Source: Front Genet. 2020 Jan 31;11:10. doi: 10.3389/fgene.2020.00010 (PMC7006602; doi:10.3389/fgene.2020.00010)
Supplement: Supplementary file 7 [file Table_4.docx]

Supplementary Table 4: Actions prioritized in the Delphi-procedure

|  | **Round 1** | | **Round 2** | |  |
| --- | --- | --- | --- | --- | --- |
| **Action** | **%** | **Top-3, n** | **%** | **Top-3, n** | **Result (round)** |
| *Roles* | | | | | |
| 1. Develop a national guideline on collaboration. | 84 | 5 | 94 | 4 | Accepted |
| 2. Make agreements on a regional level about when and who can request PGx tests. | 60 | 3 |  |  | Rejected (1) |
| *Data collection and sharing* | | | | | |
| 3. Define relevant data that should be registered and shared between health care professionals for effective use of PGx. | 95 | 5 | 100 | 6 | Accepted |
| 4. Further develop the National Link Point to enable easy exchange of PGx data between health care professionals. | 80 | 2 | 78 | 1 | Rejected (2) |
| 5. Facilitate aligned registration for the reason of adjusting a patient’s treatment regime, to monitor and evaluate effectiveness of applying PGx. | 70 | 2 |  |  | Rejected (1) |
| 6. Standardize patient data that needs to be registered with regard to PGx. | 95 | 3 | 94 | 8 | Accepted |
| 7. Adjust or develop software systems to facilitate applying PGx. | 79 | 1 |  |  | Rejected (1) |
| *Evidence* | | | | | |
| 8. Gather data on the number of prevented ineffective or adverse drug responses through PGx. | 90 | 4 | 94 | 5 | Accepted |
| 9. Monitor data on the frequency of genetic variants that are tested with PGx. | 75 | 1 |  |  | Rejected (1) |
| 10. Validate the predictive value of PGx tests through prospective or observational research. | 75 | 7 | 76 | 6 | Accepted |
| 11. Assess the cost saving of PGx test through pharmaco-economic studies. | 85 | 5 | 83 | 6 | Accepted |
| 12. Collect data on the impact on clinical outcomes by assessing the patient experience of the severity of ineffective or adverse drug response. | 80 | 2 | 78 | 4 | Accepted |
| 13. Develop aligned patient information on the benefit of PGx tests. | 85 | 2 | 94 | 7 | Accepted |
| *Reimbursement* | | | | | |
| 14. Develop aligned patient information on the costs of PGx test and the impact on their health care insurance reimbursement. | 65 | 3 |  |  | Rejected (1) |
| 15. Include PGx tests as an optional test for general practioners in their guideline. | 85 | 4 | 83 | 4 | Accepted |
| 16. Define and prioritize disease areas eligible for reimbursement based on data on clinical utility. | 75 | 3 | 56 | 3 | Rejected (2) |
